# A New Plan for Controlling Tuberculosis of Cattle

**Published:** 1902-01

**Authors:** 


### A PROPOSAL OF A NEW PLAN FOR CONTROLLING TUBERCULOSIS OF CATTLE CONSERVATIVELY.

By Columella.

The tuberculosis problem is to-day one of the most important questions before breeders of pure-bred cattle and dairymen. Evidence from every side shows that this disease is becoming more prevalent in nearly all parts of the United States, just as it is in those countries in Europe in which severe and expensive repressive measures are not in operation. Meat inspectors in slaughter-houses in the West, as well as in the East, report more tuberculosis in the cattle and swine examined by them.

The incontrovertible fact is that tuberculosis has a strong and extensive foothold in this country, and the experience of Europe furnishes us with every reason to believe that unless well-planned measures against this disease are instituted on a broad scale, and are enforced thoroughly, the conditions will in time become as bad as they are in the extensively infected countries of Europe.

This paper is not to furnish proof that tuberculosis is a source of great loss to the live-stock owner and, in some stages, of danger to consumers of milk. These points are fully covered elsewhere.[1]

It may be here stated, however, that there is no disease or influence that interferes more with the grading up of cattle in the Eastern and Central States than the losses that come from tuberculosis.[2] More good herds are ruined and more good breeders are discouraged by tuberculosis than by any other cause.

For some time it has been evident that something should be done by governmental agencies to repress this disease. The national government and many States have instituted measures with this end in view. The various regulations that are in force differ widely in their scope and effectiveness. Most of those in use by the States are tentative and experimental. That they are more or less unsatisfactory is shown by the fact that they are changed every few years, and none of them can be said to be established.[3]

Under some of the systems now in use good work is being done, but on too small a scale: all of the State operations lack scope. For example, tuberculosis is being eradicated from many individual herds in Pennsylvania; but the difficulty of keeping these herds free from tuberculosis is great on account of the fact that there remain so many tubercular herds to reinfect them. While progress is being made, it is slow.

The important question arises: Can the repression of tuberculosis be carried on more rapidly or effectively with the funds that are available? A large portion of the money allotted to every plan for the repression of tuberculosis is used in paying for tuberculous cows. In some States these cows are killed in slaughterhouses, and their flesh is used as food if the disease is not too far advanced. In other States such cows are killed in fertilizer factories or on farms, and their flesh is not used for food purposes. In

[1] Pearson and Ravenel, Bulletin No. 75, Pennsylvania Department of Agriculture; Salmon, Bulletins Nos. 32 and 33, Bureau of Animal Industry, United States Department of Agriculture; Repp, American Medicine, vol. ii., Nos. 17 and 18.

[2] "Several years ago the writer of this communication received a letter from a good citizen in the State of Iowa, asking him to name some breeder of good cattle of a certain breed from whom the inquirer might purchase some choice stock. As a result this Iowa party came to Wisconsin and purchased seven head of pure-bred stock, paying a good price for them. In taking this stock back to Iowa he carried back tuberculosis in the most virulent form it ever affected a herd in this State. He lost all of the seven animals purchased, through death by tuberculosis, and contaminated his whole herd so that he was forced to sacrifice all of the dairy animals he had. And this is but one of more than a dozen places where tuberculosis was spread by sales from a single herd of pure-bred cattle." (Prof. W. A. Henry, in Hoard's Dairyman, December 28, 1900.)

[3] For a review of these methods, see Pearson and Ravenel, Bulletin No. 75, Pennsylvania Department of Agriculture.

the former case there is some saving; in the latter case the saving is insignificant.

A great many cows discovered to be tuberculous by the use of the tuberculin test are in the early stages of the disease, and their capacity to produce calves and milk or to put on flesh is not yet impaired. Since there is no means at present to distinguish between the immediately dangerous and prospectively dangerous reacting cows, it is necessary to isolate all such cattle and destroy them or maintain them as a separate herd.

Under the plan proposed and extensively used by Professor Bang tuberculous cows are kept in separate herds, their calves are removed from contact with them soon after birth, and their milk is Pasteurized, and is sold or is used for making butter. By this means tuberculosis is being eradicated in Denmark at a comparatively rapid rate.

The Danish plan has been proposed for use in the United States. It has been used experimentally in this country in a number of instances with success so far as the repression of disease is concerned. It has been described in many speeches, reports, and papers. It has been offered to the farmers of Pennsylvania, along with some aid from the State; but it has failed to find acceptance from the owners of cattle to an extent that makes it of general service. There must be a reason for this. The reason appears to lie in the difficulty that farmers experience in disposing of the milk of a few reacting cows—because such milk has to be Pasteurized, and is feared—and in the increased labor and expense of maintaining a separate herd and enforcing the necessary precautions.

When the Bang system is proposed to the owner of reacting cows he usually says, "No, it is not worth while; give me the indemnity the State allows, and away with them." If the States were willing or could afford to thus buy and destroy all tuberculous cows no doubt the disease could in time be eradicated, but at terrific, if not impossible, cost.[1]

Cannot some way be devised to encourage farmers to adopt the Bang plan, or to otherwise stop the loss of at once destroying tuberculous cows that have not yet lost their usefulness, but which must be removed from contact with healthy cattle?

The expense and trouble of Pasteurizing milk is not great if all

1 To the statement sometimes made that "tuberculosis is a necessary evil," it may be said that it is not a necessary evil until a cow with it is brought into the herd. It does not exist in Jersey; it does not exist in many large regions of this country, nor in thousands of herds in infected districts.

of the milk is handled in the same way, and if the operation is conducted on a scale large enough to justify the purchase and economic use of modern and appropriate apparatus. The trouble of keeping and caring for a herd of tuberculous cows is not—except in reference to the calves—greater than in keeping and caring for a herd of healthy cows. The extra trouble and expense come chiefly from dividing the original herd and in caring for the two parts separately.

With the above in view, and bearing in mind conditions that are evident and need not be specifically mentioned, my proposal is as follows :

1. Let certain persons be licensed to purchase and maintain tuberculous cows and to sell such cows to persons equally licensed.

2. Let licenses for the above purpose be granted, by a board, to persons who have the intelligence, integrity, and facilities for conducting the business safely.

3. Let such licenses be free, and granted subject to certain stipulated conditions necessary to the safe conduct of the business, and subject to withdrawal when it shall be evident that these conditions are not strictly observed.

4. As part of the necessary conditions the following are suggested :

*a*. A full report on tuberculous animals purchased, sold, and on hand shall be made to a board at regular stated times by each licensee.

*b*. Tuberculous cattle shall be kept only on farms and in buildings reserved for their exclusive use.

*c*. The milk shall be Pasteurized in a way that shall be prescribed by a board or that shall be satisfactory to the expert appointed by a board.

*d*. Calves shall be removed from their dams within three days after birth, and also from the building in which tuberculous cows are kept. They shall be removed to another farm as soon as they are tested with tuberculin and are found to be healthy.

5. After adequate heating, the sale of the milk for use as food or for manufacture shall be permitted.

6. All cows going into the possession of licensees under these regulations shall be branded, tattooed, or ear-marked in some characteristic and ineffaceable way.

7. Cows so kept may be disposed of for slaughter at slaughter-houses under approved inspection when notice of intention to send for slaughter is given to the meat-inspector in charge and is acknowledged by him.

8. The cows held by licensees shall be inspected physically from time to time, and all advanced and udder cases shall be destroyed, upon the payment of moderate indemnity from the public funds.

The purpose of this whole plan is to enable herd owners to dispose of tuberculous cows at the least possible loss; in other words, to obtain for them their full value. The effect of this would be to stimulate herd owners to have their herds tested, and thus eradicate disease. At present this cannot be done without great loss. No State pays for tuberculous cows as much, on the average, as they are worth as judged by their real earning power, and no State is prepared to buy all tuberculous cows at any price.

If licenses were freely granted to all individuals applying who could meet the necessary requirements, there would be enough purchasers to ensure competition among the buyers of reacting cows, and they would bring their real market value as based on their probable earning power.

As to the use that could be made of these cows, creameries or cheese factories supplying the general trade could well afford to purchase the milk they would produce. Such milk could, after adequate heating, be certified as wholesome by a proper public officer, and there would be no valid ground for refusing to accept it, or such milk could be manufactured on the farm. In addition, some of these cows have high value for breeding, and their progeny could be reared advantageously.[1]

The most obvious apparent objection to this plan, and the one most likely to be urged by persons who are not familiar with this subject, is that it would be wrong to encourage the use and sale of diseased milk. This must be granted; but the objection shows a misconception of the plan. In the first place, nothing is added to the volume of milk from diseased cows that is now being produced. Instead of favoring the surreptitious sale of milk from diseased cows, as is now done, and entirely in the guise of milk

[1] The writer was recently consulted in the case of a very valuable herd of dairy cattle that had been entirely free from tuberculosis for several years. It happened that one or more tuberculous cows were added to this herd. Upon the next test eleven cows reacted. These were very valuable animals; all were in good condition and were highly bred; some were imported. The owner was advised to keep these cows as a separate herd. He found that he could not do so profitably, and all were killed. Only recent local lesions of slight extent were found in nine of the cows; the other two had older lesions. None of these cows could have been retained with safety in the herd: but all of them could have produced healthy calves, and some of them would probably have stood use as dairy cows for years.

from healthy cows, probably and possibly dangerous cows would be gathered together, and their milk would be rendered wholesome before it could be sold or used. In the second place, the number of tuberculous cows would be constantly reduced, and the wholesomeness of the general milk-supply would thus be improved. To insist on this objection is most irrational so long as milk from tuberculous cows is everywhere sold without the least restrictions.

Instead of encouraging the permeation of the entire cattle industry with this scourge and the contamination of all lines of the cattle trade, the infection would be directed through certain guarded channels into definite receptacles, from which it could not escape and wherein it would gradually be eradicated.

In short, the plan is to encourage the removal of tuberculous cattle from herds by paying to their owners all they are actually worth, and more than States are willing or can afford to pay; to concentrate tuberculous cattle in certain definite herds, where they can be watched and guarded closely; to so treat the products of tuberculous cattle that they can do no harm, and to thus gradually corner and eradicate the disease.

